# Letter to the editor: the effects of extracorporeal shock wave therapy in children with cerebral palsy: a systematic review

**DOI:** 10.1097/JS9.0000000000002998

**Published:** 2025-07-08

**Authors:** ZhaoJun Mei, Hujun Wang

**Affiliations:** a Luzhou Maternal and Child Health Hospital, Luzhou Second People’s Hospital, Luzhou, China; b Department of Rehabilitation, Beijing Rehabilitation Hospital, Capital Medical University, Beijing, China

*Dear Editor,*

We read with keen interest the recently published article entitled “The effects of extracorporeal shock wave therapy in children with cerebral palsy: a systematic review”^[1]^. This study presents a thorough literature retrieval strategy and categorization method, aiming to summarize the therapeutic effects of extracorporeal shock wave therapy (ESWT) in pediatric patients with cerebral palsy. As researchers with a longstanding focus on pediatric rehabilitation and movement disorder interventions, we fully acknowledge the relevance of this topic and appreciate the authors’ contributions. Nevertheless, we would like to raise several critical perspectives concerning the clinical assumptions, methodological rigor, consistency of intervention, and interpretation of results presented in the review.

The article appropriately underscores the multifaceted adverse effects of spasticity on function, participation, and quality of life in children, which form a solid rationale for therapeutic intervention. However, the review does not clearly distinguish between the neural and muscular components of spasticity, nor does it explore in depth which specific pathophysiological targets – such as neuromuscular junctions, myofascial structures, or collagen accumulation – might be modulated by ESWT. Future investigations may benefit from focusing more precisely on these subdomains. Although the review adheres to PRISMA and AMSTAR guidelines, the literature search was limited to databases such as PubMed, Cochrane Library, and PEDro. Key databases like Embase and Web of Science were not included, which may have introduced selection bias^[2]^. Regarding quality assessment, the authors utilized the PEDro scale to evaluate methodological quality; however, trials with low scores (3–4) were still included in the primary analysis. The lack of sensitivity analyses to examine the influence of these studies on the pooled effects may compromise the robustness of the conclusions. Some studies reported effect sizes as high as 14.62, which far exceed the moderate effect sizes typically observed in rehabilitation research (0.5–0.8), raising concerns about data quality and the validity of the computation methods. Especially in the absence of original datasets or clear documentation of means and standard deviations, there is a risk of overestimation. Furthermore, most included trials assessed short-term outcomes within 4–12 weeks post-treatment. Few studies provided long-term follow-up data beyond 6 months, which is crucial to evaluating whether ESWT could serve as an alternative to botulinum toxin injections or reduce the need for surgical interventions, an inherently long-term issue of structural remodeling and functional adaptation. While the review presents a generally positive stance on the potential of ESWT and even suggests its future role in spasticity management for cerebral palsy, we believe such optimism should be tempered by the current heterogeneity in intervention protocols, unclear mechanisms, and inconsistent research quality. A more cautious interpretation, emphasizing that ESWT appears preliminarily effective but requires further confirmation through rigorous randomized controlled trials, would be more appropriate.

As a non-invasive and low-risk modality, ESWT undoubtedly offers a promising avenue for managing spastic cerebral palsy. However, as highlighted in the review, substantial heterogeneity remains in terms of treatment parameters, biological mechanisms, dosage intensity, and target populations. In light of these uncertainties, we urge future studies to focus on well-designed, multicenter RCTs with extended follow-up periods to better determine the clinical utility and long-term impact of this therapy.

## Data Availability

Not applicable.

## Research registration unique identifying number (UIN)

There are no conflicts of interest.
